# Impact of active surveillance for prostate cancer on the risk of depression and anxiety

**DOI:** 10.1038/s41598-022-17224-w

**Published:** 2022-07-28

**Authors:** Davidson Sypre, Géraldine Pignot, Rajae Touzani, Patricia Marino, Jochen Walz, Stanislas Rybikowski, Thomas Maubon, Nicolas Branger, Naji Salem, Julien Mancini, Gwenaelle Gravis, Marc-Karim Bendiane, Anne-Deborah Bouhnik

**Affiliations:** 1 Department of Surgical Oncology 2, Institut Paoli-Calmettes, 232 boulevard de Sainte Marguerite, 13009 Marseille, France; 2 SESSTIM, Sciences Economiques et Sociales de la Santé et Traitement de l’Information Médicale, Institut Paoli-Calmettes, Marseille, France; 3 INSERM, IRD, SESSTIM, Sciences Economiques et Sociales de la Santé et Traitement de l’Information Médicale, ISSPAM, Equipe CANBIOS Labellisée Ligue 2019, Aix Marseille Univ, Marseille, France; 4 Medical Oncology Department, Institut Paoli-Calmettes, Marseille, France; 5 Public Health Department (BIOSTIC), APHM, Marseille, France

**Keywords:** Oncology, Urology

## Abstract

Active surveillance (AS) is a standard treatment option for low risk localized prostate cancer. However, the risk of anxiety and depression compared to other curative strategies, namely radical prostatectomy (RP) and radiotherapy (RT), is controversial. This study consisted in a French representative sample of 4174 5-years cancer survivors. Self-reported data, including quality-of-life assessment, were prospectively collected through telephone interviews. Among the 447 survivors with PC, we selected 292 patients with localized prostate cancer, T1–T2 stage, Gleason score ≤ 7 and we compared anxiety and depressive symptoms according to treatment strategy. Among patients on AS, 14.9% received curative treatment during the 5 years of follow-up. Anxiety was reported in 34.3% of cases in the AS group versus 28.6% in the RP group and 31.6% in the RT group (*p* = 0.400), while depressive symptoms were reported in 14.9% of cases in the AS group versus 10.7% in the RP group and 22.8% in the RT group (*p* = 0.770). Consumption of anxiolytics reported did not vary significantly between the 3 groups (*p* = 0.330). In conclusion, patients managed with AS for localized prostate cancer do not report more anxiety or depressive symptoms than patients managed with curative treatment, encouraging the extended use of active surveillance.

Active surveillance (AS) is a standard treatment option for low risk localized prostate cancer (PC)^1–4^.

This strategy allows men to avoid or delay treatment with surgery or radiation therapy and the related side effects that may have a potentially unfavorable impact on quality of life (QOL), without compromising cancer-specific survival at 10 years^1^.

Regardless of the treatment, anxiety and depression are the most common psychological conditions affecting cancer patients, responsible in some cases for a significant deterioration in QOL^5–7^.

In patients with PC managed by AS, intolerance of uncertainty is a predisposing trait for anxiety marked by the tendency to perceive uncertainty as threatening^8,9^. The patients may experience feelings of anxiety and distress while living with ‘‘untreated’’ cancer. The establishment of close monitoring with regular PSA testing, digital rectal examination, prostate MRI and biopsies as an integral part of AS could exacerbate perceptions of threat and therefore cause concern^10^. Yet, the management of this anxiety therefore seems essential in order to allow better patient adherence to this treatment modality^11,12^. Few studies have focused on anxiety and depressive symptoms experienced by men undergoing AS for prostate cancer.

The aim of our study was to assess the long-term impact of active surveillance on the risk of anxiety and depression compared to other curative strategies, namely radical prostatectomy (RP) and radiotherapy (RT).

## Methods

### Data source

The French VICAN5 survey was a multicenter national French prospective cohort carried out in 2015–2016 among cancer survivors, 5 years after their diagnosis of cancer. The study was approved by the national ethics committee and all patients signed an informed consent. The study methodology and data collection procedure has been detailed elsewhere^13^.

Three types of data were collected. (1) A patient questionnaire administered by phone, which included data on living conditions, Health-related Quality of Life (HR-QOL), anxiety and depression. (2) Medical and clinical data collected from healthcare teams who initiated the cancer treatment. (3) Medico-administrative data using the national health insurance database (SNIIRAM) which recovers all on their healthcare consumption since diagnosis ^14^.

### Study population

**Figure 1:**
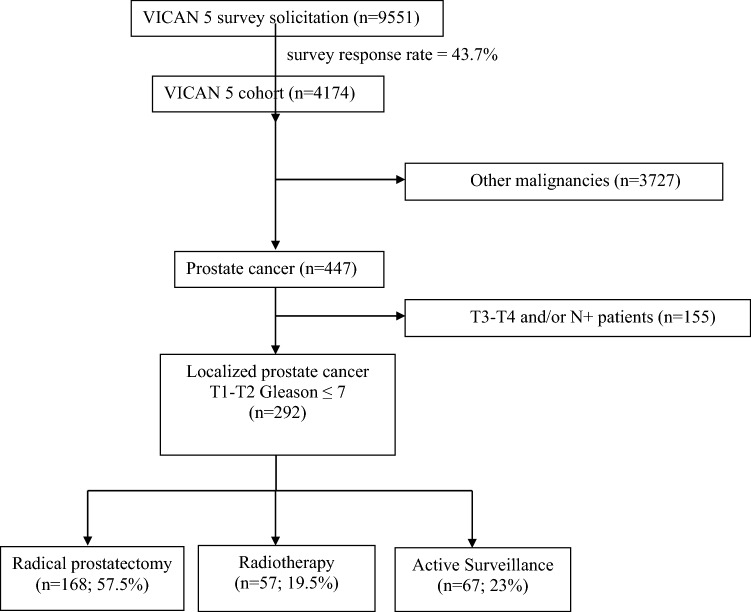
Consort diagram.

In this study, the population was restricted to men diagnosed with prostate cancer (*n* = 447). We selected patients with localized prostate cancer, stage T1–T2, Gleason score ≤ 7, treated with radical prostatectomy (RP), radiotherapy (RT) or AS, who responded to the 5-year survey (VICAN5). Patients with recurrence requiring salvage therapy were excluded. Patients receiving more than 6 months of concomitant or sequential hormone therapy were excluded. A total of 292 men met the eligibility criteria (Fig. 1).

### Primary outcomes

Anxiety and depression were measured using the Hospital Anxiety Depression scale (HAD), a validated patient-reported instrument. To create binary measures, Anxious/depressed participants were identified as those having an anxiety/depression score above 10^15^.

### Additional covariates

The consumption of anxiolytics at 5 years of the diagnosis was assessed using the national health insurance database (SNIIRAM). HR-QOL was measured using the French version of the SF12 which allow to build two health scores, the Physical Component Summary (PCS) and the Mental Component Summary (MCS)^16^.

### Statistical analyses

Chi-squared tests, Fisher exact tests, student’s t-tests were used in descriptive analyzes, depending on the type of variables. To identify the factors associated with anxiety and depression, univariate and multivariate analyzes using binary logistic regressions were performed. A sensitivity analysis (adjusting for age and treatment received) was performed by excluding patients who received delayed curative treatment after initial AS. All these analyses were performed in the STATA software program, version 17.0 (StataCorp., TX, USA).

### Ethical standards and informed consent statement.

The VICAN cohort study has been approved by the Institute of Public Health (ISP; C11-63), by the French Commission on Individual Data Protection and Public Liberties (CNIL; 911290) and by the Comité Consultatif sur le Traitement de l'Information en Matière de Recherche dans le Domaine de la Santé (CCTIRS; 11-143). The VICAN study therefore has been performed in accordance with the ethical standards laid down in the 1964 Declaration of Helsinki. All participants gave their informed consent.

## Results

### Characteristics of the population

Among the 292 patients with localized prostate cancer, T1–T2 stage, Gleason score ≤ 7, participants underwent RP alone in 57.5% of cases, RT in 19.5%, while 23% of participants had AS. Among patients on AS, 14.9% received curative treatment during the 5 years after diagnosis.

The baseline characteristics of our population are presented in Table 1. In the AS group, we found a greater distribution of urban dwellers (*p* = 0.034), a higher WHO Performance Status score (*p* = 0.003) and a lower clinical stage (*p* = 0.018) compared to the curative treatment groups. In the RT group, we observed significantly more elderly patients (*p* < 0.001), a greater number of inactive (*p* = 0.023), a significantly higher WHO Performance Status score (*p* = 0.002) and a higher proportion of clinical stage T2 (*p* = 0.023), compared to AS or RP group.

**Table 1:** Characteristics of the population according to treatment strategy.

	Global population *n* = 292	Active surveillance *n* = 67 (23%)	Radical prostatectomy *n* = 168 (57.5%)	Radiotherapy *n* = 57 (19.5%)	*p*-value*	*p*-value**
Age at VICAN 5M(SD)***	70.9 (6.7)	71.5 (7.1)	68.9 (6.1)	76.1 (5.0)	** < 0.001**	0.445
**Matrimonial status**						
Married/partner	235 (80.5)	50 (74.6)	140 (83.3)	45(78.9)	0.288	0.169
Single/divorced/separated/widower	57 (19.5)	17 (25.4)	28 (16.7)	12(21.1)		
**Education level**						
No diploma	21(7.2)	6 (9.0)	10 (6)	5 (8.8)		
Low than bachelor	153 (52.4)	36 (53.7)	81 (48.2)	36 (63.1)	0.160	0.709
Bachelor and more	118 (40.4)	25 (37.3)	77 (45.8)	16 (28.1)		
**Professional situation (*n* = 287)**						
Active	27(9.2)	7(10.4)	20 (11.9)	0		
Inactive	256(87.7)	57 (85.1)	144 (85.7)	55 (96.5)		
On sick leave or disability	4(1.4)	1(1.5)	3 (1.8)	0	**0.013**	0.846
Unknown	5(1.7)	2(3.0)	1(0.6)	2(3.5)		
**Perceived financial situation (*n* = 281)**						
Comfortable	55 (18.8)	13 (19.4)	29 (17.3)	13 (22.8)		
Getting by/must be careful	206 (70.6)	46 (68.7)	125 (74.4)	35 (61.4)	0.757	0.910
Difficult to make ends meet	20 (6.8)	5 (7.4)	11 (6.5)	4 (7.0)		
Unknown	11 (3.8)	3 (4.5)	3 (1.8)	5 (8.8)		
**Geographical area (*n* = 288)**						
Rural	98 (33.5)	30 (44.8)	53 (31.6)	15 (26.3)		
Urban	190 (65.1)	37 (55.2)	114 (67.9)	39 (68.4)	0.092	**0.034**
Unknown	4 (1.4)	0	1 (0.5)	3 (5.3)		
**WHO performance status (*n* = 168)**						
0	49 (16.8)	19 (28.4)	26 (15.5)	4 (7.0)		
1	114 (39.0)	18 (26.9)	64 (38.1)	32 (56.1)	**0.002**	**0.003**
2	5 (1.7)	2 (2.9)	2 (1.2)	1 (1.8)		
Unknown	124 (42.5)	28 (41.8)	76 (45.2)	20 (35.1)		
**T stage (*n* = 222)**						
Tx	5 (1.7)	1 (1.5)	4 (2.4)	0		
T1	75 (25.7)	27 (40.3)	31 (18.4)	17 (29.8)	**0.023**	**0.018**
T2	142 (48.6)	27 (40.3)	86 (51.2)	29(50.9)		
Unknown	70 (24.0)	12 (17.9)	47 (28.0)	11 (19.3)		
**Gleason score (*n* = 225)**						
< 7	137 (46.9)	37 (55.2)	76 (45.2)	24 (42.1)		
= 7	88 (30.1)	20 (29.9)	46 (27.4)	22 (38.6)	0.376	0.471
Unknown	67 (23.0)	10 (14.9)	46 (27.4)	11 (19.3)		

*p*-value calculated without taking into account missing data.

*p-value comparing the three groups (AS vs RP vs RT).

**p-value comparing AS vs RP+RT.

***M (SD): Mean (Standard Deviation).

Significant values are bold.

### Anxiety

Among patients managed with AS, 34.3% (23/67) reported anxiety, compared to 28.6% (48/168) in the RP group, and 31.6% (18/57) in the RT group, with no significant difference (*p* = 0.400). Consumption of anxiolytics reported did not vary significantly between the 3 groups (*p* = 0.330). The results are presented in Table 2.

**Table 2:** Results.

	Global population (*n* = 292)	Active surveillance * n* = 67 (23%)	Radical Prostatectomy *n* = 168 (57.5%)	Radiotherapy *n* = 57 (19.5%)	*p* value*	*p* value**
**Depressive symptoms (*n* = 287)**						
No	246 (83.4)	55 (82.1)	149 (88.7)	42 (73.7)		
Yes	41 (14.0)	10 (14.9)	18 (10.7)	13 (22.8)	0.059	0.770
Missing	5 (1.7)	2 (3.0)	1 (0.6)	2 (3.5)		
**Anxiety (*n* = 286)**						
No	197 (67.5)	42 (62.7)	119 (70.8)	36 (63.1)		
Yes	89 (30.5)	23(34.3)	48 (28.6)	18 (31.6)	0.573	0.400
Missing	6 (2.0)	2 (3.0)	1 (0.6)	3 (5.3)		
**Consumption of anxiolytics**						
None	168 (57.5)	35 (52.2)	99 (58.9)	34 (59.7)		
Only after the diagnosis	92 (31.5)	26 (38.8)	51 (30.4)	15 (26.3)	0.581	0.330
Consumption before and after the diagnosis	32 (11.0)	6 (9.0)	18 (10.7)	8 (14.0)		

*p*-value calculated without taking into account missing data.

*p-value comparing the three groups (AS vs RP vs RT).

**p-value comparing AS vs RP + RT.

### Depressive symptoms

Among patients managed with AS, 14.9% (10/67) reported depressive symptoms, compared to 10.7% (18/168) in the RP group, and 22.8% (13/57) in the RT group, with no significant difference (*p* = 0.770). The results are presented in Table 2.

### Predictive factors of anxiety and depression in patients with localized prostate cancer

A financial situation perceived as critical or difficult was significantly associated with depressive symptoms (*p* = 0.002) and anxiety (*p* = 0.044) (Table 3).

**Table 3:** Factors associated with depression and anxiety: univariate analyzes.

	Depressive symptoms (*n* = 287)		Anxiety (*n* = 286)	
	No	Yes	No	Yes
	*n* = 246 (85.7%)	*n* = 41 (14.3%)	*n *= 197 (68.9%)	*n* = 89 (31.1%)
Age at VICAN 5M(SD)*	***P = *0.303**		***P = *0.501**	
	70.7 (6.7)	71.8 (6.6)	70.6 (6.8)	71.2 (6.5)
Professional situation	***P = *0.394**		***P = *0.827**	
Active	25 (10.2)	2 (5.0)	18 (9.1)	9 (10.2)
Inactive and others	221 (89.8)	38 (95.0)	179 (90.9)	79 (89.8)
Perceived financial situation	***P* = 0.002**		***P = *0.044**	
Comfortable	47 (19.1)	8 (19.5)	41 (20.8)	14 (15.7)
Getting by/must be careful	182 (74.0)	24 (58.5)	144 (73.1)	62 (69.6)
Difficult to make ends meet	12 (4.9)	8 (19.5)	9 (4.6)	11 (12.4)
Unknown	5 (2.0)	1 (2.5)	3 (1.5)	2 (2.3)
WHO performance status	***P* = 0.251**		***P* = 0.715**	
0	44 (17.9)	5 (12.2)	35 (17.8)	14 (15.7)
≥ 1	97 (39.4)	21 (51.2)	79 (40.1)	39 (43.8)
Unknown	105 (42.7)	15 (36.6)	83 (42.1)	36 (40.5)
T stage	***P* = 0.232**		***P* = 0.379**	
Tx	4 (1.6)	1 (2.4)	4 (2.0)	1 (1.2)
T1	65 (26.4)	7 (17.1)	54 (27.4)	18 (20.2)
T2	115 (46.8)	25 (61.0)	92 (46.7)	48 (53.9)
Unknown	62 (25.2)	8 (19.5)	47 (23.9)	22 (24.7)
Gleason score	***P* = 0.207**		***P* = 0.115**	
< 7	120 (48.8)	16 (39.0)	90 (45.7)	46 (51.7)
= 7	69 (28.0)	15 (36.6)	64 (32.5)	20 (22.5)
Unknown	57 (23.2)	10 (24.4)	43 (21.8)	23 (25.8)
Physical quality of life M(SD)*	***P* < 0.001**		***P* = 0.017**	
	48.7 (8.1)	42.6 (9.4)	48.6 (8.3)	46.0 (8.8)
Mental quality of life M(SD)*	***P* < 0.001**		***P* < 0.001**	
	50.3 (7.6)	37.3 (9.3)	51.2 (7.1)	42.4 (10.0)
Significant fatigue	***P* < 0.001**		***P* < 0.001**	
No	187 (76.0)	16 (39.0)	155 (78.7)	48 (53.9)
Yes	59 (24.0)	25 (61.0)	42 (21.3)	41 (46.1)

*p*-value calculated without taking into account missing data.

***M (SD): Mean (Standard Deviation).

Significant values are bold.

There was a significant association between depression/anxiety and a decreased mental HR-QOL (*p* < 0.001 and *p* < 0.001 respectively), a decreased physical HR-QOL (*p* < 0.001 and *p* = 0.017 respectively), and reported fatigue (*p* < 0.001 and *p* < 0.001 respectively).

According to our multivariate analyses, no significant association between depressive symptoms and anxiety and the treatment received was observed (Table 4).

**Table 4:** Factors associated with depression and anxiety: multivariate analysis.

	Depressive symptoms Ref. (No)		Anxiety Ref. (No)	
	aOR	95% CI	aOR	95% CI
Age at VICAN 5	0.98	0.92–1.05	1.03	0.98–1.08
**Treatment strategy**				
AS	1		1	
Radical prostatectomy	0.64	0.24–1.75	0.70	0.34–1.43
Radiotherapy	1.70	0.55–5.29	0.85	0.34–2.10
**WHO performance status**				
0	1		1	
≥ 1	1.32	0.45–3.89	1.02	0.48–2.15
**Professional situation**				
Active	1		1	
Inactive and others	2.98	0.35–25.74	0.64	0.21–1.96
**Perceived financial situation**				
Comfortable	1		1	
Getting by/must be careful	0.62	0.24–1.59	1.16	0.54–2.47
Difficult to make ends meet	2.79	0.70–11.12	2.89	0.83–10.10

95% CI 95% confidence intervals.

Ref., Reference; aOR, Adjusted odds ratios.

The results do not change by excluding patients who received delayed curative treatment after initial AS (14.9%) (Supplementary Table 1 and 2).

## Discussion

In our study, we showed that men with prostate cancer on AS, 5 years after their diagnosis of cancer, didn’t present an increased risk of anxiety or depression compared to patients treated by RP or RT, which is also reflected by the absence of overconsumption of anxiolytics. These results are consistent with those of the previously published ProtecT study, which assessed anxiety and depression using the Hospital Anxiety and Depression Scale (HADS)^17^. In the current literature regarding AS patients, the majority of the studies found low levels of anxiety, prostate cancer-specific anxiety, and depression^17–21^. Punnen et al. longitudinally studied anxiety and depression in 679 men who underwent RP or AS within 1 year, and between 1 and 3 years from baseline. Anxiety symptoms were measured using the General Anxiety Disorder scale 7 (GAD-7), distress was ascertained using the Distress Thermometer and Depressive symptoms were assessed using the Patient Health Questionnaire (PHQ-9). No difference in prevalence rates of depression, anxiety, and distress over time was noted , with < 5% of patients exhibiting moderate or high levels of depression or anxiety in both groups^18^. Van den Bergh et al. reported that patients with low-risk PC who chose AS showed low anxiety and distress from the time of diagnosis up to 9 months. Significant decreases were seen between 2.4 and 9.2 months after diagnosis in mean scores of general anxiety (STAI-6) (*p* = 0.016), prostate cancer-specific anxiety (MAX-PC), fear of progression subscale (*p* = 0.005), and self-estimated disease progression risk (*p* = 0.049)^19^. Men with prostate cancer on AS exhibit low general and illness-specific anxieties, relayed by Jake Anderson et al. A high percentage of men had low levels of general state anxiety as measured by the HADS-A (86%) and trait anxiety as measured by the STAI-T (77%). For illness-specific anxieties, 87% of men reported low levels of prostate cancer-related anxiety and 92% reported low levels of fear of recurrence^20^. In a study conducted by Marzouk et al., men undergoing active surveillance did present a moderate risk of cancer specific anxiety, which was 29% risk of reporting cancer specific anxiety within the first year. Moreover, anxiety significantly decreased with time^21^.

In the patients on AS, some risk factors of anxiety and depression have been identified in the literature. Greater intolerance of uncertainty and moderate/severe urinary symptoms have been described as risk factors in patients on AS, as shown by Tan et al.^22^. Other risk factors as neurotic personality score seemed to be an important determinant of anxiety and distress in men on AS^23^. In our study, we identified new factors significantly associated with anxiety and depression in patients with localized prostate cancer, in particular a financial situation perceived as critical or difficult. Less surprisingly, the other parameters of HR-QOL, namely mental or physical HR-QOL and reported fatigue, were also associated with anxiety and depression.

Strengths of our study are the prospective, population-based and nationwide design, which facilitates generalizability. Moreover, we ensure long-term follow-up with a larger sample at 5 years, and a high questionnaire response rate which increased the precision and internal validity. Finally, the evaluation of the consumption of anxiolytics is an interesting parameter that has not been usually evaluated previously.

Several limitations of the present study require discussion. First, the active surveillance protocol was not standardized, as it was left to the discretion of the urologist, assuming regular PSA monitoring and repeat biopsies according to national guidelines. Similarly, the criteria for treatment choice were not detailed. These limitations are inherent in the design of the VICAN observational survey. A second limitation is that this research used self-report measures by phone, which may limit the objectivity of participant responses and induce a risk of information bias. In the end, the distribution of patients in each group is unequal, without randomization, which can induce a patient selection bias. Although the overall response rate is quite high in this survey (43% of patients with prostate cancer at diagnosis), we do not know if the response rate was similar across the different treatment groups (AS versus other), especially for patients presenting with symptoms of depression or anxiety, and this could induce a nonresponse bias.

## Conclusions

Patients managed by AS for localized prostate cancer do not report more anxiety or depression than patients managed by curative treatment, encouraging the extended use of AS for patients with low-risk localized prostate cancer.

## Supplementary Information

Supplementary Information 1.

Supplementary Information 2.

## Data Availability

Data are available if required.
