# Supplementary material for: Impact of active surveillance for prostate cancer on the risk of depression and anxiety
Source: Sci Rep. 2022 Jul 28;12:12889. doi: 10.1038/s41598-022-17224-w (PMC9334351; doi:10.1038/s41598-022-17224-w)
Supplement: Supplementary file 1 — Supplementary Information 1. [file 41598_2022_17224_MOESM1_ESM.docx]

**Supplementary Table 1. Anxiety, depression and consumption of anxiolytics according to treatment strategy (without patients who had treatment after AS)**

|  | **Global population**  **n=280** | **Active surveillance**  **n=55 (19.6%)** | **Radical prostatectomy**  **n=168 (60.0%)** | **Radiotherapy**  **n=57 (20.4%)** | **p-value*** | **p-value**** |
| --- | --- | --- | --- | --- | --- | --- |
| **Depressive symptoms (n=276)** |  |  |  |  | 0.056 | 0.613 |
| No | 236 (84.3) | 45 (81.8) | 149 (88.7) | 42 (73.7) |  |  |
| Yes | 40 (14.3) | 9 (16.4) | 18 (10.7) | 13 (22.8) |  |  |
| Missing | 4 (1.4) | 1 (1.8) | 1 (0.6) | 2 (3.5) |  |  |
| **Anxiety (n=275)** |  |  |  |  | 0.487 | 0.308 |
| No | 189 (67.5) | 34 (61.8) | 119 (70.8) | 36 (63.2) |  |  |
| Yes | 86 (30.7) | 20 (36.4) | 48 (28.6) | 18 (31.5) |  |  |
| Missing | 5 (1.8) | 1 (1.8) | 1 (0.6) | 3 (5.3) |  |  |
| **Consumption of anxiolytics** |  |  |  |  | 0.794 | 0.595 |
| None | 162 (57.9) | 29 (52.7) | 99 (58.9) | 34 (59.7) |  |  |
| Only after the diagnosis | 86 (30.7) | 20 (36.4) | 51 (30.4) | 15 (26.3) |  |  |
| Consumption before and after the diagnosis | 32 (11.4) | 6 (10.9) | 18 (10.7) | 8 (14.0) |  |  |

^p-value calculated without taking into account missing data^

^* p-value comparing the three groups (AS vs RP vs RT)^

^**p-value comparing AS vs RP+RT^
