# Supplementary material for: Impact of active surveillance for prostate cancer on the risk of depression and anxiety
Source: Sci Rep. 2022 Jul 28;12:12889. doi: 10.1038/s41598-022-17224-w (PMC9334351; doi:10.1038/s41598-022-17224-w)
Supplement: Supplementary file 2 — Supplementary Information 2. [file 41598_2022_17224_MOESM2_ESM.docx]

**Supplementary Table 2. Sensitivity analysis: factors associated with depression and anxiety by removing patients who had treatment after AS (n=280)**

|  | **Depressive symptoms**  **Ref. (No)** | | | **Anxiety**  **Ref. (No)** | | |
| --- | --- | --- | --- | --- | --- | --- |
|  | **aOR** | | **95% CI** | **aOR** | | **95% CI** |
| **Age at VICAN 5** | 0.96 | | 0.90 – 1.04 | 1.02 | | 0.97 – 1.08 |
| **Treatment strategy** |  | | |  | | |
| AS | 1 | | | 1 | | |
| Radical prostatectomy | 0.61 | | 0.21 – 1.73 | 0.63 | | 0.30 – 1.35 |
| Radiotherapy | 1.80 | | 0.54 – 5.93 | 0.81 | | 0.31 – 2.09 |
| **WHO Performance Status** |  | | |  | | |
| 0 | 1 | | | 1 | | |
| ≥1 | 1.11 | 0.37 – 3.30 | | 0.96 | | 0.45 – 2.08 |
| **Professional situation** |  | | |  | | |
| Active | 1 | | | 1 | | |
| Inactive and others | 3.13 | | 0.34 – 28.39 | 0.69 | 0.20 – 2.44 | |
| **Perceived financial situation** |  | | |  | | |
| Comfortable | 1 | | | 1 | | |
| Getting by/ Must be careful | 0.58 | | 0.22 – 1.51 | 1.11 | | 0.52 – 2.40 |
| Difficult to make ends meet | 3.08 | | 0.75 – 12.65 | 3.23 | | 0.89 – 11.70 |

^Ref.: Reference; aOR: Adjusted Odds Ratios^

^95% CI: 95% confidence intervals^
